# P-2083. Outcomes of Upper Gastrointestinal Bleeding in African American Patients with HIV: A Nationwide Study

**DOI:** 10.1093/ofid/ofaf695.2247

**Published:** 2026-01-11

**Authors:** Mathangi Murali, Diviya Bharathi Ravikumar, Barath Prashanth Sivasubramanian, Dency Dineshbhai Mavani, Krishna Sai Kiran Sakalabaktula, Anusha Endreddy, Falaknaaz Mubassirhusen Saiyad, Heer Pareshbhai Shah, Jay Patel, Karthik Basumani, Neha Nanditha Adepu, Uzer Abdulaziz Memon, Naveen Yellappa, Rutul Dalal, Raghavendra Tirupathi

**Affiliations:** Government Erode Medical College and Hospital, Chennai, Tamil Nadu, India; ESIC Medical College and Postgraduate Institute of Medical Science and Research, Chennai, Tamil Nadu, India; University of Texas Health San Antonio, San Antonio, Texas; Smt. NHL Municipal Medical College, Ahmedabad, Gujarat, India; Government General Hospital, Kakinada, Kakinada, Andhra Pradesh, India; Alluri Sitarama Raju Academy of Medical Sciences, Eluru, Andhra Pradesh, India; Smt. NHL Municipal Medical College, Ahmedabad, Gujarat, India; Smt. NHL Municipal Medical College, Ahmedabad, Gujarat, India; B.J. Medical College, Ahmedabad, Gujarat, India; ESIC Medical College and Postgraduate Institute of Medical Science and Research, Chennai, Tamil Nadu, India; Osmania Medical College, Hyderabad, Telangana, India; Smt. NHL Municipal Medical College, Ahmedabad, Gujarat, India; Geisinger Commonwealth School of Medicine, Scranton, Pennsylvania; Medical Director, Penn State Health (Eastern Region), Penn State Health St. Joseph Medical Center, Pennsylvania, USA, Lancaster, Pennsylvania; Keystone Health, Chambersberg, Pennsylvania

## Abstract

**Background:**

In the United States, African Americans (AAs) experience a higher HIV-related mortality compared to American Whites (AWs) (20.1 vs 3.1 per 100,000). In HIV, upper gastrointestinal bleeding (UGIB) showed a low prevalence of 1-14%, but these patients require urgent hospitalization. Racial disparities in HIV have been previously studied, but there remains a gap in understanding their impact on UGIB. We aim to evaluate the racial disparities affecting mortality in HIV patients with UGIB.

**Figure:**
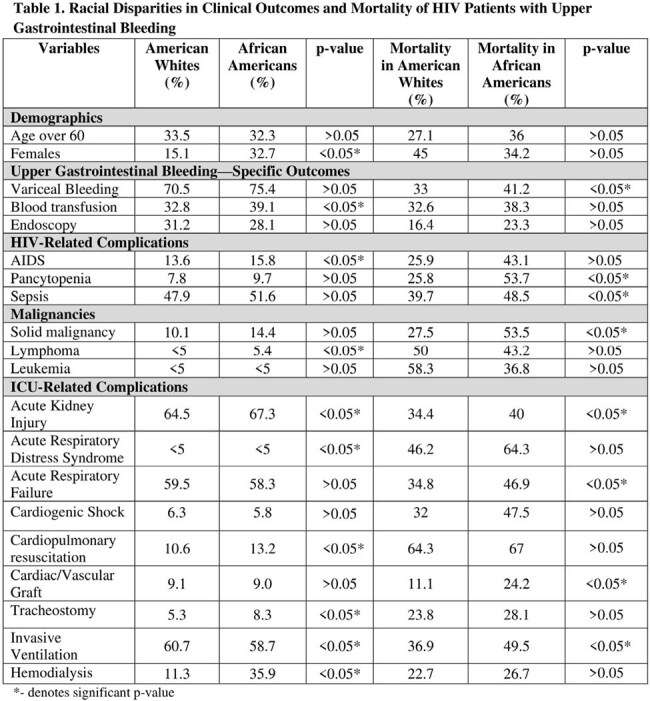

**Figure:**
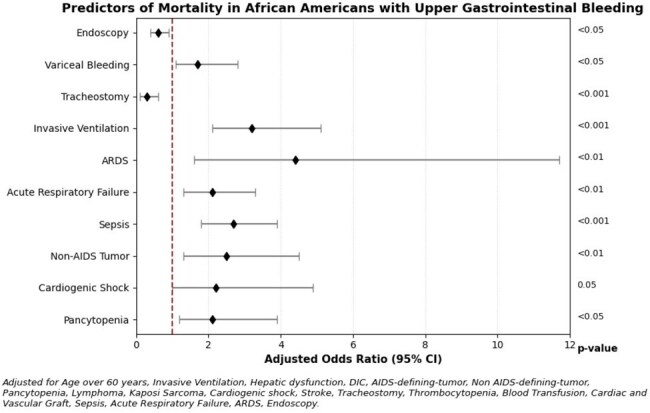

**Methods:**

A retrospective analysis was conducted using the National Inpatient Sample database (2016-2021). Adults aged ≥ 18 with an ICD-10 code of HIV and UGIB who required ICU-level care were included. ICU-level care was identified by the presence of shock, acute kidney injury requiring hemodialysis, or the requirement of invasive ventilation. Multivariate analysis was used to determine mortality risk by adjusting for sociodemographic factors and comorbidities. A p ≤ 0.05 was adopted.

**Results:**

A total of 6,923 patients required ICU-level care. The admission rates were similar between AAs and AWs (17.3% vs 17.9%); however, mortality was higher in AAs than AWs (37.1% vs 29.9%; OR 1.5; p = 0.01). AAs had higher mortality with variceal bleeding (41.2% vs 33%; OR 1.6, p = 0.01), AIDS defining illness (45.1% vs 30%; OR 1.8, p = 0.05), and sepsis (48.5% vs 39.7%; OR 1.5, p < 0.05). AAs had lower rates of receiving palliative care services than AWs (15.7% vs 18.9%; OR 0.8, p = 0.1). In the AA cohort, several complications were significantly associated with mortality (p < 0.05). These included: pancytopenia (OR 2.1), cardiogenic shock (OR 2.2), ARDS (OR 4.4), invasive ventilation (OR 3.2), and sepsis (OR 2.7). Endoscopy (OR 0.6) showed lower mortality risk, emphasizing the benefits of procedural care in this high-risk population.

**Conclusion:**

Among HIV patients with upper gastrointestinal bleeding, African American individuals experienced higher mortality than American White individuals. These findings underscore the need for equitable healthcare access, culturally sensitive interventions, and targeted strategies to identify and address barriers in this population.

**Disclosures:**

All Authors: No reported disclosures

